# Mechanical Behavior Investigation of 4H-SiC Single Crystal at the Micro–Nano Scale

**DOI:** 10.3390/mi11010102

**Published:** 2020-01-17

**Authors:** Peng Chai, Shujuan Li, Yan Li, Lie Liang, Xincheng Yin

**Affiliations:** School of Mechanical and Precision Instrument Engineering, Xi’an University of Technology, Xi’an 710048, China; chaipeng@stu.xaut.edu.cn (P.C.); dreamtrainll@163.com (L.L.); albertyinxc@163.com (X.Y.)

**Keywords:** 4H-SiC single crystal, critical indentation depth, plastic–brittle transition, hardness, modulus, deformation

## Abstract

In this paper, theoretical models of the critical indentation depth and critical force on brittle materials using cleavage strength and contact theory are proposed. A Berkovich indenter is adopted for nanoindentation tests on a 4H-SiC single crystal sample to evaluate its mechanical behaviors. The stages of brittle material deformation (elastic, plastic, and brittle) can be characterized by the load versus indentation depth curves through the nanoindentation test. The curve of the elastic deformation stage follows the Hertz contact theory, and plastic deformation occurs at an indentation depth of up to 10 nm. The mechanism of 4H-SiC single crystal cracking is discussed, and the critical indentation depth and critical force for the plastic–brittle transition are obtained through the occurrence of the pop-in point. This shows that the theoretical results have good coherence with the test results. Both the values of the elastic modulus and hardness decrease as the crack length increases. In order to obtain more accurate mechanical property values in the nanoindentation test for brittle materials such as SiC, GaN, and sapphire, an appropriate load that avoids surface cracks should be adopted.

## 1. Introduction

With the rapid development of the modern semiconductor industry, silicon-based semiconductors are quickly approaching their limit according to Moore’s Law [1], and an important direction for the semiconductor industry will be in looking for a substitute for silicon. In this process, silicon carbide (SiC) became of interest to researchers in recent decades [2,3,4,5,6,7]. SiC is an ideal material for manufacturing semiconductor devices on account of its excellent physical and chemical properties, such as its wide bandgap, high critical breakdown field strength, high electron saturation rate, high thermal conductivity and stability, and chemical inertness [8,9]. Compared with silicon devices, the applied range of SiC devices is wider, especially in high pressure and high temperature environments [9,10,11]. It has been reported that the large diameter growth of SiC, such as 150 mm [12] and 200 mm [13], has been successful, and this provides the necessary conditions for the large-scale application of SiC devices. Moreover, SiC is also an excellent material with which to manufacture a quantum computer chip in the future [14]. However, as a brittle and hard material, brittle fracture and crack propagation can easily occur on the machining surface of SiC. The conventional machining process of SiC wafers includes slicing, lapping, and polishing, and the mechanisms of those process are reliant on the behavior of single diamond grit.

Nanoindentation testing refers to a technology using a diamond indenter (Berkovich, cone, pyramid, and sphere), for which the mechanical properties are given, to determine another material’s mechanical properties by means of indentation. Essentially, it is a process of driving an indenter into the material surface and investigating the mechanical behavior (hardness *H*, modulus *E*, creep compliance *C*, etc.) of the material based on the response of the indenter [15,16]. In 1992, Oliver and Pharr proposed a systematic analytical method of indentation hardness and elasticity modulus based on nanoindentation [17]. Through more than twenty years of development, this method has become a common criterion, and has been adopted extensively by commercial indentation apparatus manufacturers. Meanwhile, the international testing standards of nanoindentation have been formulated [18], and reports on SiC with nanoindentation technology are growing. Nawaz et al. [19] investigated the elastic-plastic response and plastic deformation of 6H-SiC single crystal using the depth sensing nanoindentation technique. Matsumoto et al. [20] clarified the mechanical properties of 4H-SiC single crystal by means of nanoindentation with a Berkovich indenter, and studied the deformation process. Ma et al. [21] researched the nanoindentation behavior of polycrystalline 3C-SiC thin films exhibiting columnar microstructures by acoustic emission signal monitoring and time-frequency spectrum analysis. Lu et al. [22] studied 6H-SiC substrate characteristics by means of nanoindentation and found that the C face is easier to remove than the Si face during mechanical planarization machining. Datye et al. [23] evaluated the fracture toughness and plastic behavior of 6H-SiC single crystal by nanoindentation. Pang et al. [24] conducted indentation experiments on 6H-SiC single crystal, and the results showed that classical crystal plasticity theory can be reliably applied in predicting the plastic deformation of ceramic at small scales. Goal et al. [25] analyzed displacement controlled quasistatic nanoindentations on single crystal 4H-SiC, and an analytical stress analysis was carried out to calculate the theoretical shear strength and tensile stress.

**Figure 1:**
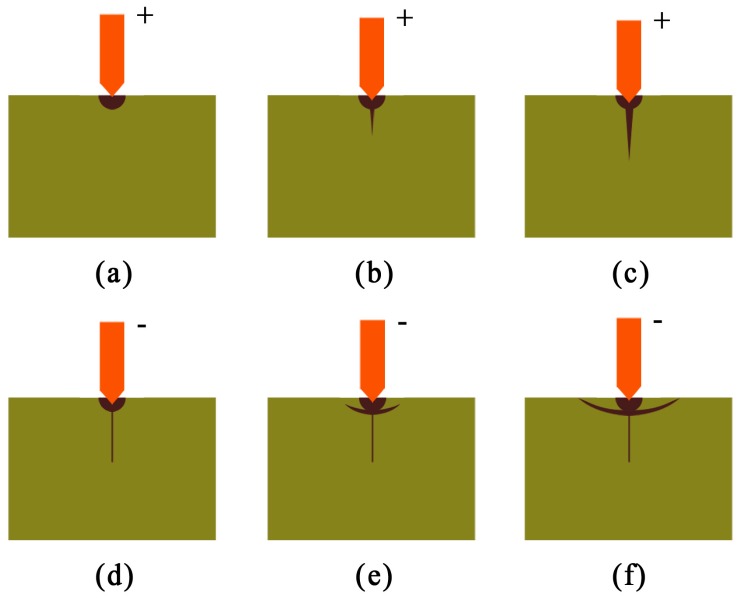
Microfracture model for brittle materials: (**a**) Initial loading; (**b**) Critical zone formation; (**c**) Stable crack growth; (**d**) Initial unloading; (**e**) Residual-stress cracking; (**f**) Complete unloading.

Researchers have concluded that there is plastic deformation in the micro–nano machining process of brittle materials, such that machined surface damage will be avoided and high surface quality can be achieved [26,27,28,29]. The most widely adopted model to explain brittle material deformation is that developed by Lawn et al. [30], shown in Figure 1. Figure 1a shows a zone of elastic and plastic deformation around the contact point during the initial loading process. Figure 1b–f shows the process of crack generation and propagation, and it is clear that these deformations are brittle. Thus, for nanoindentation, there is a critical indentation depth for the plastic–brittle transition. When the loading depth of the indenter is more than the critical indentation depth, a subsurface crack will appear on the sample.

With the development of nanotechnology, the load sensitivity and displacement sensitivity of nanoindentation instruments have been improved greatly, and this provides convenient conditions for the investigation of material characteristics at the nanoscale. For the purpose of this paper, analyses of the deformation caused by the pressure of Berkovich indenter on the commercial wafer surface of 4H-SiC single crystal are carried out. The critical indentation depth and critical force for plastic–brittle transition are obtained from the theoretical models and analyses of the loading curves. The changing law of elasticity modulus and hardness with the increase of the indentation depth is discussed. These provide important technical parameters for the plastic region process of 4H-SiC single crystal.

## 2. Experimental Procedure and Methods

### 2.1. Materials and Preprocess

The commercially available 4H-SiC single crystal wafer, with a 2 inch diameter and 500 micrometer thickness, supplied by the Shanghai Institute of Optics and Mechanics Chinese Academy of Sciences (Shanghai, China) and grown using the Physical Vapor Transport (PVT) method, was used in this experiment. The smooth surface of the wafer is the (0001) Si face and the surface roughness is less than 1 nm after a polishing treatment. The wafer was diced into samples with a size of 10 mm × 10 mm by a laser cutting machine (HGLaser LCC0130-CO2, HGTECH, Wuhan, China). Before testing, acetone was used to decontaminate the sample surface. A Berkovich indenter made of single crystal diamond and a standard fused quartz sample for calibration of the function area was used in this study.

### 2.2. Experimental Design

**Figure 2:**
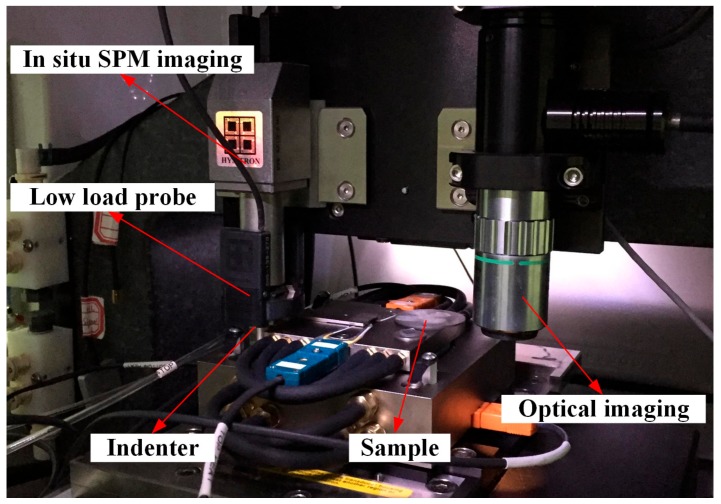
Core component of the TI 950.

All tests were conducted on a nanomechanical test instrument (TI 950 Triboindenter, Hysitron, Minneapolis, MN, USA) at room temperature, whose load sensitivity was less than 30 nN and displacement sensitivity was less than 0.2 nm. In situ scanning probe microscope (SPM) images could be obtained through dual piezo scanners of TI 950. The core component of the instrument is shown in Figure 2. The nanoindentation process on the surface of 4H-SiC single crystal is simple and includes three stages: (1) the loading stage, (2) the holding stage, and (3) the unloading stage. In the first stage, the indentation loading was applied at a constant rate until the set maximum load, and the loading time was 10 s. In the holding stage, the indenter at the maximum load was held for 5 s. In the unloading stage, the indenter was unloaded linearly and the unloading time was 10 s. The maximum load varied in a range of 0.4–9 mN in the low load module, and the maximum load changed in a range of 100–1000 mN in the high load module. The thermal drift was kept below ±0.1 nm/s for all indentations.

A focussed ion beam milling combined with scanning electron microscopy (FIB-SEM) system (Helios NanoLab 600i, FEI, Hillsboro, OR, USA) was used to measure and evaluate the pressed surface deformation on the 4H-SiC sample immediately after the nanoindentation tests.

### 2.3. Determination of Hardness and Modulus

**Figure 3:**
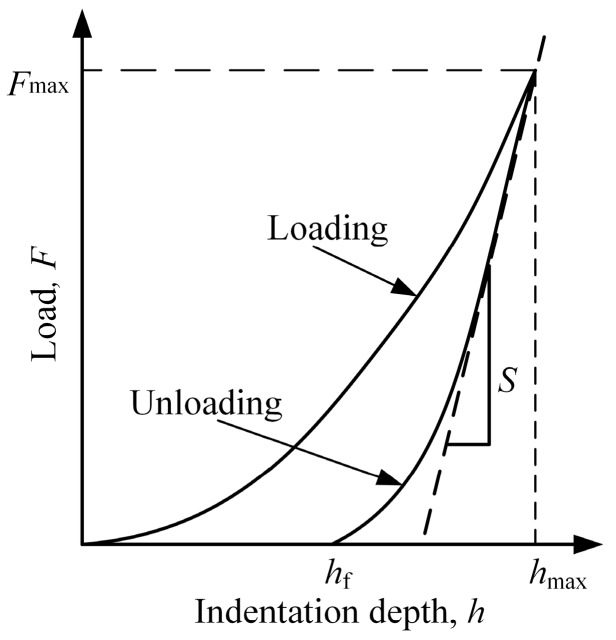
Typical load-indentation depth curve.

A typical load-displacement curve is shown in Figure 3. During the loading procedure, elastoplasticity deformation occurred on the surface of the specimen, and only part of the elastic deformation can be restored at the time of unloading. As shown in Figure 3, *h_f_* refers to the depth of residual indentation after complete unloading, and *S* is the contact stiffness, which can be figured out by calculating the slope below a fifth from the top of the unloading curve (*S* = d*F*/d*h*).

The hardness and elasticity modulus can be calculated using the Oliver–Pharr method by the following equations [17]:

(1) $$H=\frac{F_{max}}{A_{p}},$$

(2) $$\frac{1}{E_{r}}=\frac{1-{\left(\nu_{s}\right)}^{2}}{E_{s}}+\frac{1-{\left(\nu_{i}\right)}^{2}}{E_{i}},$$

where *F_max_* is the peak indentation load, *A_p_* is projected contact area of the indenter and specimen, *E_r_* is the reduced elastic modulus, *ν_s_* is the Poisson’s ratio of the specimen, *ν_i_* is the Poisson’s ratio of the indenter, *E_s_* is the elasticity modulus of the specimen, and *E_i_* is the elasticity modulus of the indenter.

The reduced elastic modulus, *E_r_*, is determined from the contact stiffness, *S*, and the projected contact area, *A_p_*, as follows:

(3) $$E_{r}=\frac{\sqrt{\pi }}{2\beta }\frac{S}{\sqrt{A_{P}}},$$

where the *β* parameter is a correction factor.

The projected contact area of the indenter and specimen, *A_p_*, is calculated from:

(4) $$A_{p}(h_{c})=24.5h_{c}^{2}+C_{1}h_{c}^{1}+C_{2}h_{c}^{1/2}+C_{3}h_{c}^{1/4}+\cdot \cdot \cdot +C_{8}h_{c}^{1/128},$$

where *C*_1_ through *C*_8_ are constants accounting for tip rounding and other departures from the ideal shape.

The contact depth, *h_c_*, is calculated from:

(5) $$h_{c}=h_{max}-\varepsilon \frac{F}{S},$$

where *h_max_* is the peak indentation depth, and *ε* is a geometric constant (for the Berkovich indenter, *ε* = 0.75).

### 2.4. Determination of the Critical Indentation Depth for the Plastic–Brittle Transition

**Figure 4:**
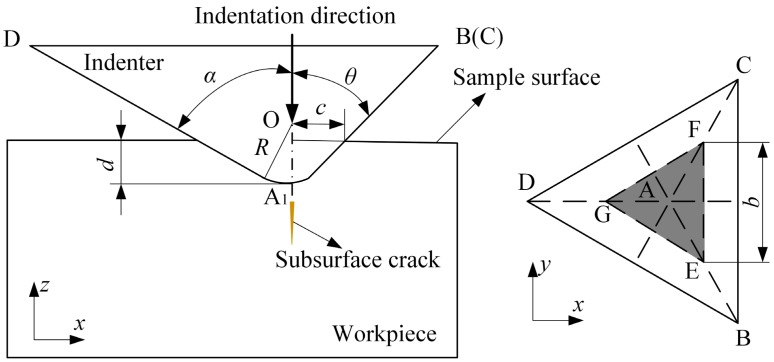
Indentation model.

Figure 4 shows the indentation model with a Berkovich indenter. Based on the microfracture model for brittle materials, with increasing load, subsurface cracks will occur when the maximum stress at the bottom of the indenter reaches the cleavage strength. The cleavage strength can be expressed by [31]:

(6) $$\sigma_{c}=\frac{1}{2}\sqrt{\frac{E\gamma }{a}},$$

where *E* is the elastic modulus, *γ* is the surface energy per unit area, and *a* is the interplanar spacing. The maximum stress is [32]:

(7) $$P_{0}=\frac{3}{2}P_{m},$$

where *P_m_* is the uniform pressure. The critical condition of brittle materials for plastic–brittle transition during the nanoindentation process is:

(8) $$\sigma_{c}=P_{0}.$$

The indentation load is:

(9) $$F=P_{m}A,$$

where *A* is the cross-sectional area of the indenter corresponding to the indentation depth; that is, the shaded area shown in Figure 4, and it can be calculated according to the geometric relationships of the Berkovich indenter:

(10) $$A=\frac{3\sqrt{3}}{4}{\left(d+\frac{R}{\sin\alpha }-R\right)}^{2}\tan^{2}\alpha ,$$

where *d* is the indentation depth, *R* is the nose radius of the Berkovich indenter, and *α* is an angle between the edge line and the centerline (for the Berkovich indenter, *α* = 77.3°).

The load versus indentation depth curve presents a tendency of exponential function in the process of loading, and the load can be expressed as [33]:

(11) $$F=ad^{n},$$

where *a* is a coefficient of the material property, and *n* is a coefficient of the indenter shape.

From the above equations, the critical indentation depth and critical force of brittle materials for the plastic–brittle transition can be obtained.

## 3. Results Analysis and Discussion

### 3.1. Elastic and Plastic Deformation of Nanoindentation

**Figure 5:**
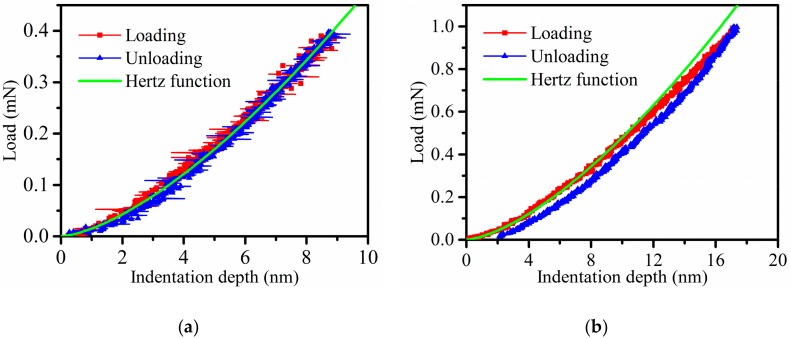
They are load versus indentation depth curves for elastic deformation and plastic deformation of indentations: (**a**) the elastic deformation (maximum load of 0.4 mN) and (**b**) the plastic deformation (maximum load of 1 mN).

When the peak load reaches 0.4 mN and 1 mN, respectively, the load versus indentation depth curves of indentations performed on the 4H-SiC single crystal sample are shown in Figure 5. Figure 5a shows that the unloading curves overlap the loading curves, hence, only the elastic deformation occurred on the sample surface during nanoindentation. The research by Chang et al. [34] indicated that the tip of the Berkovich indenter can be considered as spherical when the contact depth is less than 20 nm. The nose radius of the Berkovich indenter of these tests was taken as 1160 nm, the method in Chai’s work [35] is adopted, and nanoindentation tests were conducted with a standard fused quartz sample. According to the Hertz contact theory, the normal force of the contact between a rigid sphere and an elastic half-space body can be described by:

(12) $$F=\frac{4}{3}E_{r}R^{0.5}h^{1.5},$$

where *R* is the sphere radius and *h* is the indentation depth. The value of *E_r_* is determined in Section 3.3. The load versus indentation depth curves follow the Hertz contact theory in elastic deformation of 4H-SiC single crystal. Figure 5b shows that the unloading curve and loading curve are separated, and the residual indentation depth after removal of the force is 2.1 nm. It is demonstrated that irreversible plastic deformation happened on the sample surface. The loading curve follows the Hertz contact theory when the depth is less than 10 nm, but with the increase of depth, the loading curve no longer follows this theory. For one thing, the contact surface between the Berkovich indenter and sample become more complicated, and for another, the plastic deformation begins to play a major role. Furthermore, the smooth loading curves reveal good elastic and plastic deformations. Although the research by Han et al. [36] indicated that the load versus indentation strain curve over single crystal silicon would have pop-in point at the stage of plastic deformation, the load curves shown in Figure 5 have no obvious pop-in point. The reason is that the strain rate of the specimen above the reference is high, and the dislocation glide velocity is fast, but in contrast, these tests are quasi-static tests.

### 3.2. Critical Indentation Depth for the Plastic–Brittle Transition

**Figure 6:**
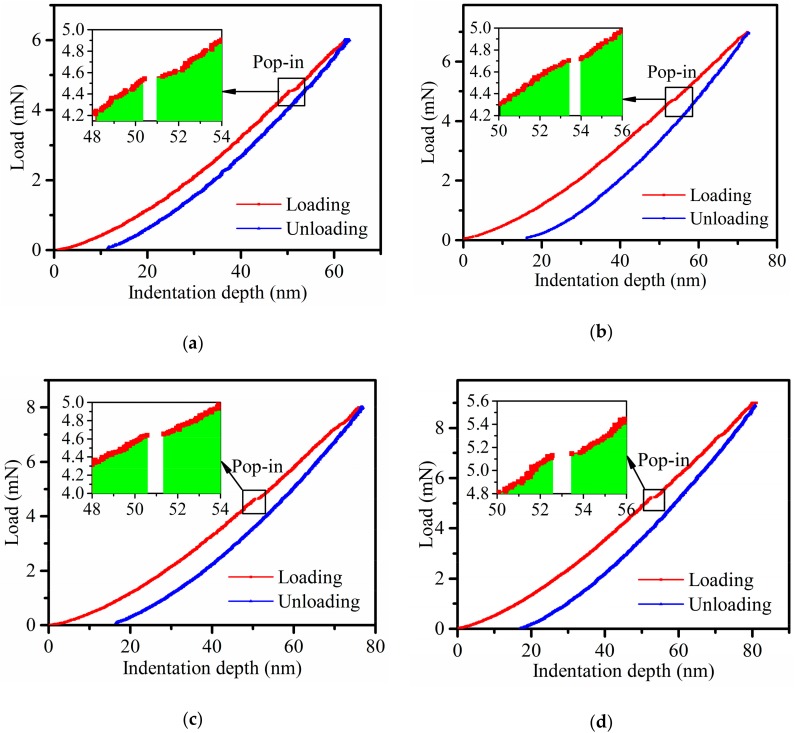
The load versus indentation depth curves for brittle deformation of indentations: (**a**) the maximum load of 6 mN, (**b**) the maximum load of 7 mN, (**c**) the maximum load of 8 mN, and (**d**) the maximum load of 9 mN.

Figure 6 shows the load versus indentation depth curves in the case of a maximum indentation load of 6 mN, 7 mN, 8 mN, and 9 mN, and all the load curves reflect the pop-in point. According to the Zener–Mott–Stroh dislocation pile-up theory [37], the source generates edge dislocation loops which pile up at barriers in the glide plane when the resolved shear stress reaches a certain value. Under the shear stress, these dislocations form a dislocation pile-up cluster and the stress concentration at the barrier results in the nucleation of microcracks. If the formation of a crack is reflected on the load versus indentation depth curve, the pop-in point occurs. The critical indentation depth for the plastic–brittle transition and pop-in load are shown in Table 1. Test results indicate that the average critical indentation depth of 4H-SiC single crystal with a Berkovich indenter with a nose radius of 1160 nm is 51.7 nm, and the corresponding average pop-in load is 4.8 mN. According to the theoretical models of the critical load and critical indentation depth of brittle material for the plastic–brittle transition described in Section 2.4, the elastic modulus is obtained through the discussion in Section 3.3, considering the anisotropy of crystal material the surface energy *γ* of 4H-SiC single crystal is $3.0\pm_{1.0}^{1.6}$ J/m^2^ [38], the interplanar spacing of *a* is 0.3079 nm, the correlation coefficient *a* of material property based on fitting of the loading-displacement curve is 3.79 × 10^8^ N/m^1.5^ (average result of four curves), for which R-square is 0.9998, and the correlation coefficient *n* of the Berkovich indenter shape is 1.5. On this basis, the theoretical value of the critical indentation depth of 4H-SiC single crystal for the plastic–brittle transition is 20.8 to 64 nm, and the critical load is 1.1 to 6.1 mN. The test result is within the range of theoretical values, proving the feasibility of this method.

### 3.3. The Influence of Cracks on the Modulus and Hardness

**Figure 7:**
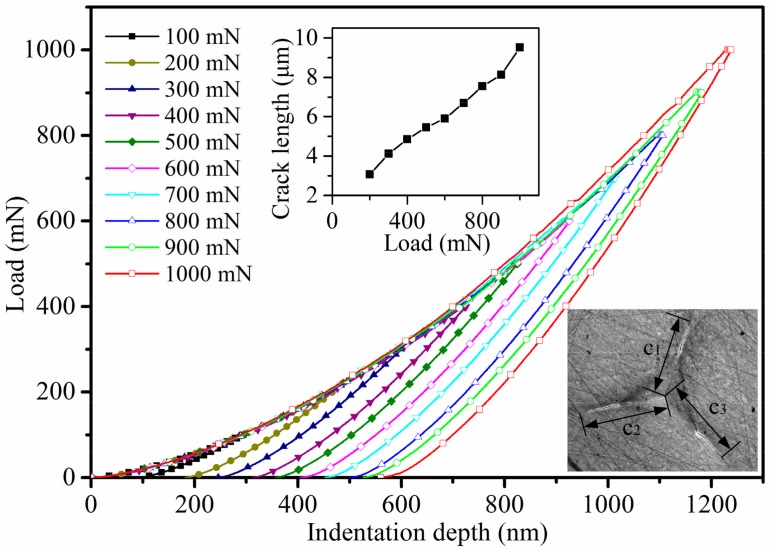
Load versus indentation depth curves with a maximum load range from 100–1000 mN for 4H-SiC single crystal samples (the small chart shows the crack length-load curve and the measuring method of the crack length is shown in the lower right corner).

**Figure 8:**
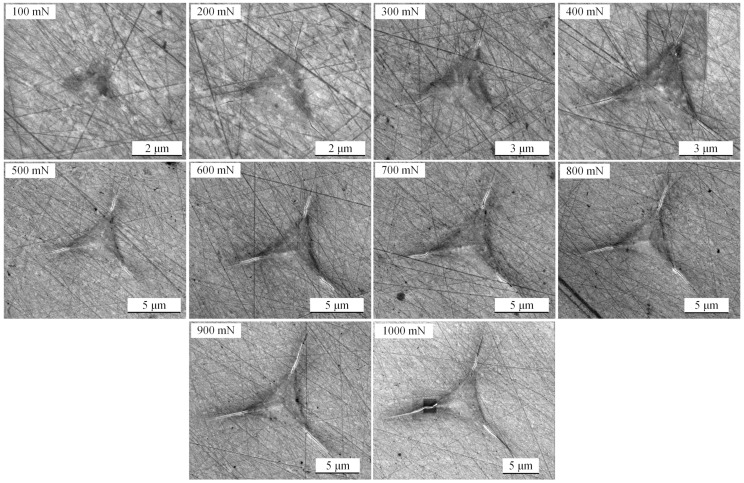
Scanning electron microscopy (SEM) images of indentations at a peak load of 100 mN, 200 mN, 300 mN, 400 mN, 500 mN, 600 mN, 700 mN, 800 mN, 900 mN, and 1000 mN on the 4H-SiC single crystal sample surface.

Figure 7 shows the load versus indentation depth curves of 10 indentations using the high load module with a maximum load range from 100 to 1000 mN for 4H-SiC single crystal samples, and Figure 8 shows the SEM images for the corresponding indentations. The relationship between load and average length (from indentation center to crack tip) of the three cracks is shown in the small chart in Figure 7.

**Figure 9:**
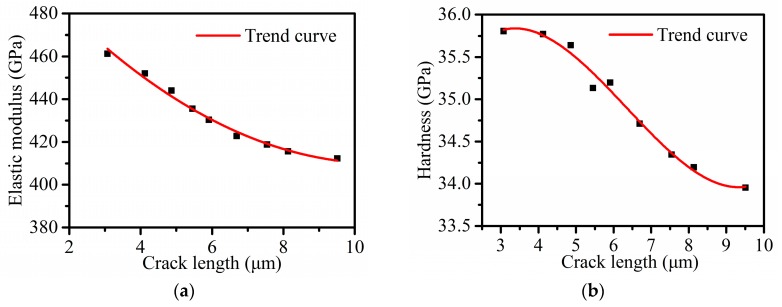
Mechanical properties as a function of crack length: (**a**) elastic modulus as a function of crack length and (**b**) hardness as a function of crack length.

It can be seen from Figure 8 that surface cracks are rarely observed when the maximum load is 100 mN, they can be observed obviously when the maximum load is more than 200 mN, and they enlarge approximately linearly with the increase of the maximum load. There are a lot of scratches on the sample surface of 4H-SiC single crystal after polishing. They have a certain inducing effect on the initial crack, but the influence becomes smaller and smaller with the growth of the crack. The crack length in three directions is different because of the anisotropy of 4H-SiC single crystal [39]. The Poisson’s ratio of 4H-SiC single crystal is 0.2, the Poisson’s ratio of diamond is 0.07, the elastic modulus of diamond is 1140 GPa [40], and the *β* parameter of the Berkovich indenter is 1.05 [41]. According to the Oliver–Pharr method, the elastic modulus and hardness of the average results of five tests are shown in Figure 9. The values of the elastic modulus decreased from 461.3 GPa to 412.3 GPa (10.62% reduction) as the crack length increased from 3.07 μm to 9.51 μm, and the values of hardness also decreased from 35.81 GPa to 33.95 GPa (5.19% reduction) with the same crack length. The elastic modulus value of 4H-SiC with no crack is close to the values found by other researchers using different measurement methods, as shown in Table 2. Researchers often explain this phenomenon by the material properties changing with the increase of the indentation depth through the size effect, and the most widely used model to explain this phenomenon is that developed by Nix and Gao using the concept of geometrically necessary dislocations [42,43,44,45,46]. However, according to the discussion in Section 3.1, the brittle deformation plays a leading role in these indentations leading to surface cracks. It is not enough to explain changing trends of elastic modulus and hardness in Figure 9 using dislocation. From Equations (1) and (3), we consider that the crack extension increases the projected contact area *A_p_*, and the reduced modulus and hardness decrease with the increase of the projected contact area. In order to obtain more accurate mechanical property values in nanoindentation tests for brittle materials like SiC, an appropriate load for avoiding surface cracks should be adopted.

## 4. Conclusions

The nanoindentation tests were conducted on 4H-SiC single crystal samples with a Berkovich indenter in a nanomechanical test system. The deformation characteristics and mechanical properties of 4H-SiC single crystal were studied by load versus indentation depth curves and SEM images of indentations using the microfracture model and Hertz contact theory. The following conclusions can be drawn from this study:

1. The stages of brittle material deformation (elastic, plastic, and brittle) can be characterized by the load versus indentation depth curves through the nanoindentation test. The curve of the elastic deformation stage follows the Hertz contact theory, and the plastic deformation occurs in nanoindentation at an indentation depth of up to 10 nm.
2. The crackling mechanism of 4H-SiC single crystal is discussed and the theoretical models of critical indentation depth and critical force for the plastic–brittle transition are proposed using cleavage strength theory and contact theory. The test results were obtained through the occurrence of the pop-in point, and the theoretical results show good agreement with the test results.
3. Both the values of elastic modulus and hardness decrease as the crack length increases, because the crack extension increases the projected contact area *A_p_*. In order to obtain more accurate mechanical property values in nanoindentation tests for brittle materials like SiC, an appropriate load for avoiding surface cracks should be adopted.

## Figures and Tables

**Table 1:** The critical indentation depth and critical force for the plastic–brittle transition.

Max Load (mN)	Indentation Depth (nm)	First Pop-in Depth (nm)	First Pop-in Load (mN)
6	62	50.3	4.56
7	72	53.4	4.72
8	77	50.6	4.75
9	82	52.6	5.16
Average	-	51.7	4.80

**Table 2:** The elastic modulus of 4H-SiC measured by different methods.

Face	Modulus (GPa)	Method	Reference
(0001)	434	Ultrasonic pulse	[47]
(1100)	503.7	Cantilever	[48]
Unknown	448.2	Ab initio calculations	[49]
Unknown	480	Dynamic method	[50]

## References

[1] Hussain A.M. (2016). Extending Moore’s Law for Silicon CMOS Using More-Moore and More-than-Moore Technologies. Ph.D. Thesis.

[2] Pushpakaran B.N., Subburaj A.S., Bayne S.B., Mookken J. (2016). Impact of silicon carbide semiconductor technology in Photovoltaic Energy System. Renew. Sustain. Energy Rev..

[3] Ando T., Fu X.-A. (2019). Materials: Silicon and beyond. Sens. Actuators A Phys..

[4] Phan H.P., Zhong Y., Nguyen T.K., Park Y., Dinh T., Song E., Vadivelu R.K., Masud M.K., Li J., Shiddiky M.J.A. (2019). Long-Lived, Transferred Crystalline Silicon Carbide Nanomembranes for Implantable Flexible Electronics. ACS Nano.

[5] Nguyen T., Dinh T., Foisal A.R.M., Phan H.P., Nguyen T.K., Nguyen N.T., Dao D.V. (2019). Giant piezoresistive effect by optoelectronic coupling in a heterojunction. Nat. Commun..

[6] Nguyen T.-K., Phan H.-P., Dinh T., Dowling K.M., Foisal A.R.M., Senesky D.G., Nguyen N.-T., Dao D.V. (2018). Highly sensitive 4H-SiC pressure sensor at cryogenic and elevated temperatures. Mater. Des..

[7] Nagy R., Niethammer M., Widmann M., Chen Y.C., Udvarhelyi P., Bonato C., Hassan J.U., Karhu R., Ivanov I.G., Son N.T. (2019). High-fidelity spin and optical control of single silicon-vacancy centres in silicon carbide. Nat. Commun..

[8] Neudeck P.G. (2001). Silicon Carbide Electronic Devices, Encyclopedia of Materials: Science and Technology.

[9] Shcherban N.D. (2017). Review on synthesis, structure, physical and chemical properties and functional characteristics of porous silicon carbide. J. Ind. Eng. Chem..

[10] Goel S. (2014). The current understanding on the diamond machining of silicon carbide. J. Phys. D Appl. Phys..

[11] Hui Zhang L.M.T. (2011). Efficiency Impact of Silicon Carbide Power Electronics for Modern Wind Turbine Full Scale Frequency Converter. IEEE Trans. Ind. Electron..

[12] Masumoto K., Kudou C., Tamura K., Nishio J., Ito S., Kojima K., Ohno T., Okumura H. (2013). Growth of silicon carbide epitaxial layers on 150-mm-diameter wafers using a horizontal hot-wall chemical vapor deposition. J. Cryst. Growth.

[13] Kimoto T. (2016). Bulk and epitaxial growth of silicon carbide. Prog. Cryst. Growth Charact. Mater..

[14] Madar R. (2004). Silicon carbide in contention. Nature.

[15] Fischer-Cripps A.C. (2006). Critical review of analysis and interpretation of nanoindentation test data. Surf. Coat. Technol..

[16] Hu C., Li Z. (2015). A review on the mechanical properties of cement-based materials measured by nanoindentation. Constr. Build. Mater..

[17] Oliver W.C., Pharr G.M. (1992). An improved technique for determining hardness and elastic modulus using load and displacement sensing indentation experiments. J. Mater. Res..

[18] Lucca D.A., Herrmann K., Klopfstein M.J. (2010). Nanoindentation: Measuring methods and applications. Cirp Ann..

[19] Nawaz A., Mao W.G., Lu C., Shen Y.G. (2017). Mechanical properties, stress distributions and nanoscale deformation mechanisms in single crystal 6H-SiC by nanoindentation. J. Alloys Compd..

[20] Matsumoto M., Harada H., Kakimoto K., Yan J.W. (2016). Study on Mechanical Properties of Single-Crystal Silicon Carbide by Nanoindentation. Adv. Mater. Res..

[21] Ma X.G., Komvopoulos K., Bogy D.B. (2004). Nanoindentation of polycrystalline silicon-carbide thin films studied by acoustic emission. Appl. Phys. Lett..

[22] Lu J., Luo Q., Xu X., Huang H., Jiang F. (2017). Removal mechanism of 4H- and 6H-SiC substrates (0001 and 0001^−^) in mechanical planarization machining. Proc. Inst. Mech. Eng. Part B J. Eng. Manuf..

[23] Datye A., Schwarz U., Lin H.-T. (2018). Fracture Toughness Evaluation and Plastic Behavior Law of a Single Crystal Silicon Carbide by Nanoindentation. Ceramics.

[24] Pang K.H., Tymicki E., Roy A. (2018). Indentation in single-crystal 6H silicon carbide: Experimental investigations and finite element analysis. Int. J. Mech. Sci..

[25] Goel S., Yan J., Luo X., Agrawal A. (2014). Incipient plasticity in 4H-SiC during quasistatic nanoindentation. J. Mech. Behav. Biomed. Mater..

[26] Goel S., Luo X., Comley P., Reuben R.L., Cox A. (2013). Brittle–ductile transition during diamond turning of single crystal silicon carbide. Int. J. Mach. Tools Manuf..

[27] Lee S.H. (2012). Analysis of ductile mode and brittle transition of AFM nanomachining of silicon. Int. J. Mach. Tools Manuf..

[28] Goel S., Luo X., Agrawal A., Reuben R.L. (2015). Diamond machining of silicon: A review of advances in molecular dynamics simulation. Int. J. Mach. Tools Manuf..

[29] Antwi E.K., Liu K., Wang H. (2018). A review on ductile mode cutting of brittle materials. Front. Mech. Eng..

[30] Lawn B.R., Swain M.V. (1975). Microfracture beneath point indentations in brittle solids. J. Mater. Sci..

[31] Op Het Veld A.J., Veldkamp J.D.B. (1970). Mechanical and microscopical investigation of SiC whiskers. Fibre Sci. Technol..

[32] Johnson K.L. (1985). Contact Mechanics.

[33] Cheng Y.-T., Cheng C.-M. (2004). Scaling, dimensional analysis, and indentation measurements. Mater. Sci. Eng. R Rep..

[34] Chang L., Zhang L. (2009). Mechanical behaviour characterisation of silicon and effect of loading rate on pop-in: A nanoindentation study under ultra-low loads. Mater. Sci. Eng. A.

[35] Chai P., Li S., Li Y. (2019). Modeling and Experiment of the Critical Depth of Cut at the Ductile-Brittle Transition for a 4H-SiC Single Crystal. Micromachines.

[36] Han J., Sun J., Xu S., Song D., Liu H., Han Y., Fang L. (2018). Deformation mechanisms at multiple pop-ins under spherical nanoindentation of (1 1 1) Si. Comput. Mater. Sci..

[37] Lawn B. (1993). Fracture of Brittle Solids.

[38] Wells G.H., Hopf T., Vassilevski K.V., Escobedo-Cousin E., Wright N.G., Horsfall A.B., Goss J.P., O’Neill A.G., Hunt M.R.C. (2014). Determination of the adhesion energy of graphene on SiC(0001) via measurement of pleat defects. Appl. Phys. Lett..

[39] Goel S., Stukowski A., Luo X., Agrawal A., Reuben R.L. (2013). Anisotropy of single-crystal 3C–SiC during nanometric cutting. Model. Simul. Mater. Sci. Eng..

[40] Field J.E., Telling R.H. (1999). The Young Modulus and Poisson Ratio of Diamond.

[41] Díez-Pascual A.M., Gómez-Fatou M.A., Ania F., Flores A. (2015). Nanoindentation in polymer nanocomposites. Prog. Mater. Sci..

[42] Voyiadjis G., Yaghoobi M. (2017). Review of Nanoindentation Size Effect: Experiments and Atomistic Simulation. Crystals.

[43] Peng Z., Gong J., Miao H. (2004). On the description of indentation size effect in hardness testing for ceramics: Analysis of the nanoindentation data. J. Eur. Ceram. Soc..

[44] Bull S.J., Page T.F., Yoffe E.H. (1989). An explanation of the indentation size effect in ceramics. Philos. Mag. Lett..

[45] Zhu T., Bushby A., Dunstan D. (2008). Size effect in the initiation of plasticity for ceramics in nanoindentation. J. Mech. Phys. Solids.

[46] Nix W.D., Gao H. (1998). Indentation size effects in crystallinematerials: A law for strain gradient plasticity. J. Mech. Phys. Solids.

[47] Kitahara H., Noda Y., Yoshida F., Nakashima H., Shinohara N., Abe H. (2001). Mechanical Behavior of Single Crystalline and Polycrystalline Silicon Carbides Evaluated by Vickers Indentation. J. Ceram. Soc. Jpn..

[48] Nguyen T.-K., Phan H.-P., Dinh T., Han J., Dimitrijev S., Tanner P., Md Foisal A.R., Zhu Y., Nguyen N.-T., Dao D.V. (2017). Experimental Investigation of Piezoresistive Effect in p-Type 4H–SiC. IEEE Electron Device Lett..

[49] Konstantinova E., Bell M.J.V., Anjos V. (2008). Ab initio calculations of some electronic and elastic properties for SiC polytypes. Intermetallics.

[50] Islam M.M., Huang C.F., Zhao F. (2012). Single-Crystal SiC Resonators by Photoelectrochemical Etching. Mater. Sci. Forum.

